# AN OLDER CARER, BUT STILL A CHILD: UNEXPECTED IDENTITIES OF OLDER ADULTS CARING FOR A NEAR-CENTENARIAN PARENT

**DOI:** 10.1093/geroni/igz038.2277

**Published:** 2019-11-08

**Authors:** Oscar Riberio, Typhanie Macedo, Liliana Sousa

**Affiliations:** 1 University of Aveiro - Department of Education and Psychology | Center for Health Technology and Services Research (CINTESIS.UA), Aveiro, Portugal; 2 University of Aveiro - Department of Education and Psychology, Aveiro, Portugal

## Abstract

According to the National Alliance for Caregiving (2015), the typical definition of an older adult caregiver points to a 79-year-old white female who cares for a close relative due to a long-term physical condition; in such cases the care-recipient is often a spouse, an adult child or a sibling, but not a parent. This cross-sectional qualitative study explores the experience of a group of fifteen children in their 70s who are main care-providers of their parents (mean age 98; range 95-105); it focuses on their overall caregiving experience with a particular emphasis on how they feel being still a child at such an advanced age. In-depth interviews were conducted and analyzed for recurrent themes using thematic analysis. Main findings revealed that although being in an overall positive experience, often socially exalted (proudness of having a parent alive/being in an unique situation), none had expected to be holding the identity of a child at their current age. These children’s views of their ageing self were strongly embedded in their caregiving role, and some reported losing awareness of their own age and a mixture of feelings of being simultaneously a child, a parent and a grandparent. Role captivity (late life prison) and disregarding self-care shaped their current situation in terms of caregiving burden and altered life plans as older adults. This study adds to the limited available knowledge on very old caregiving dyads, and raises awareness on how personal and family identity may be shaped in older age by unforeseen family dynamics.

